# Modeling and Simulation of Dynamic Recrystallization Microstructure Evolution for GCr15 Steel Using the Level Set Method

**DOI:** 10.3390/ma18020342

**Published:** 2025-01-14

**Authors:** Xuewen Chen, Mingyang Liu, Yisi Yang, Yahui Si, Zheng Zhou, Xudong Zhou, Dongwon Jung

**Affiliations:** 1 School of Materials Science and Engineering, Henan University of Science and Technology, Luoyang 471023, China; 220320020232@stu.haust.edu.cn (M.L.); 15838510562@163.com (Y.Y.); siyahui@stu.haust.edu.cn (Y.S.); 220320020243@stu.haust.edu.cn (Z.Z.); syuuzhou@mail.haust.edu.cn (X.Z.); 2 Department of Mechanical Engineering, Jeju National University, 102 Daehak-Ro, Jeju-si 63243, Republic of Korea

**Keywords:** GCr15 bearing steel, level set, dynamic recrystallization, microstructure evolution

## Abstract

The microstructure of metallic materials plays a crucial role in determining their performance. In order to accurately predict the dynamic recrystallization (DRX) behavior and microstructural evolution during the hot deformation process of GCr15 bearing steel, a microstructural evolution model for the DRX process of GCr15 steel was established by combining the level set (LS) method with the Yoshie–Laasraoui–Jonas dislocation dynamics model. Firstly, hot compression tests were conducted on GCr15 steel using the Gleeble-1500D thermal simulator, and the hardening coefficient *k*_1_ and dynamic recovery coefficient *k*_2_ of the Yoshie–Laasraoui–Jonas model were derived from the experimental flow stress data. The effects of temperature, strain, and strain rate on DRX behavior and grain size during the hot working process of GCr15 steel were investigated. Through secondary development of the software, the established microstructural evolution model was integrated into the DIGIMU^®^ software. Metallographic images were imported in situ to reconstruct its initial microstructure, enabling GCr15 steel DRX microstructure finite element simulation of the hot compression process. The predicted mean grain size and flow stress demonstrated a strong correlation and excellent agreement with the experimental results. The results demonstrate that the established DRX model effectively predicts the evolution of the DRX fraction and average grain size during the hot forging process and reliably forecasts DRX behavior.

## 1. Introduction

High-carbon chromium alloy steel, GCr15, is a typical bearing steel widely used in the bearing industry due to its excellent processability, high wear resistance, long fatigue life, high elastic limit, superior fracture toughness, and other advantageous properties [1,2]. It is recognized as one of the most widely used steel grades in the bearing industry. The mechanical properties and service life of metal materials after hot deformation largely depend on the evolution of their microstructure [3,4]. The dynamic recrystallization (DRX) behavior of a material plays a crucial role in shaping its microstructure and influencing its mechanical properties. DRX can effectively control the performance of forgings by refining the grains and controlling the final microstructure of the forgings [5]. Additionally, DRX is known to soften metals that have undergone work-hardening stages, thereby improving their plasticity and fatigue resistance [6]. It is regarded as having important engineering application value for controlling the internal structure of metal materials, improving plastic forming capacity, and optimizing hot working technology [7]. Therefore, studying the behavior of the DRX process of GCr15 steel and improving its mechanical properties is crucial.

In the past decades, a large number of scholars around the world have investigated the DRX modeling of metals and alloys and their mechanisms in the hot forging process using a combination of theoretical and experimental methods [8,9,10]. The academic community generally recognizes two mechanisms for DRX: continuous DRX (CDRX) and discontinuous DRX (DDRX). During CDRX, the recrystallized grains are formed in a slow and continuous process. This grain formation is attributed to the gradual recombination of dislocations into cells or subgrains, with an increasing misorientation angle between subgrains [11]. On the other hand, in DDRX, the driving force for nucleation is the difference in dislocation density between grains [12]; when wavy grain boundaries are present, relatively dislocation-free regions appear at the boundaries, leading to the separation of new recrystallized grains from adjacent grains. These grains then continue to grow at the grain boundaries, significantly reducing the local dislocation density between grains and ultimately resulting in material softening [8].

With the rapid improvement of computer power, numerical simulation techniques can simulate grain growth (GG) and DRX processes more accurately [13,14,15]. Through numerical simulation, researchers and engineers can predict microstructure evolution, grain boundary migration, and other behaviors more easily. This understanding facilitates taking advantage of the relationship between the mechanical properties and microstructure of materials, providing optimized process parameters for specific product performance requirements. The existing models for studying DRX behavior can generally be divided into three main categories: phenomenological models, mean-field models, and full-field models. Among phenomenological models, the most common are the Avrami relationship and the JMAK model. The Avrami relationship correlates the global recrystallization fraction during hot deformation with time and temperature [16,17,18]. The JMAK model, on the other hand, aims to describe the relationships among global recrystallization fraction, mean grain size, and time–temperature parameters [19,20,21]. These models typically fit experimental data to mathematical equations or statistical models to describe DRX behavior but do not account for the influence on the material’s microstructure. After this, to describe the impact of grain size and dislocation density on the material’s microstructure, researchers introduced the mean-field model method, which reduces microstructure to a mean value for n grains and describes the DRX behavior by calculating the velocity of each grain with respect to the mean grain [22,23,24]. However, the mean-field model cannot describe the topology of grains and deal with large deformation; therefore, the assumptions related to the average grain curvature or nucleation location limit the applicability of the model [25,26].

To gain a deeper understanding of the DRX mechanism, researchers have further proposed the full-field (FF) modeling approach. This approach overcomes many of the limitations of mean-field models. The FF model simulates the microstructural evolution of materials at the mesoscale, using a representative volume element (RVE) to solve for the microstructural evolution. It considers the microstructural topology at the polycrystalline scale and the interactions between adjacent grains, significantly improving the applicability of the model [27]. The advent of FF finite element simulation enables more accurate simulation of DRX phenomena with Minimized Central Processing Unit (CPU) resources and enables the modeling of local microstructural features, such as abnormally large grains. There are two main categories of FF models: discrete and continuous. Among them, the Monte Carlo (MC) method, Vertex method, and Cellular Automata (CA) method are discrete full-field models. The MC [28] and CA [29] methods are widely used due to their simplicity and high computational efficiency. However, due to the stochastic nature of the unit grid simulation of the microstructure, they cannot accurately reflect the changes in grain boundary curvature. Difficulties also arise in handling multiphase deformation and grain boundary evolution [30]. Some researchers have developed the Vertex method [31], also known as the Front-Tracking method, which tracks the microstructure based on vertices. It offers a potential solution to the issue of grain boundary curvature. However, this method becomes complex when dealing with events such as the appearance of new grains or the disappearance of grains. The phase-field (PF) method and LS method are continuous full-field models. The advantage of the PF method is that it uses the phase field function to describe the microstructure implicitly and avoids the problem of tracking the interface [32]. However, the PF method has limitations, such as its inability to perform parallel computations efficiently, leading to higher computational costs and longer time requirements.

Compared to other methods, the LS method represents grain boundaries in the form of an implicit function, naturally handling complex grain boundary shapes and topological changes (such as grain merging or splitting). It can be easily coupled with equations describing physical mechanisms, such as dislocation density gradients and grain boundary migration kinetics, enabling accurate simulation of microstructure evolution. The LS method is suitable for studying grain evolution at the mesoscale and can be extended to large-scale DRX behavior prediction through coupling with mean-field models. The grain evolution results obtained using the LS method provide an intuitive visualization of grain morphology, grain boundary positions, and their dynamic changes, offering clear and valuable data for in-depth studies of DRX behavior. Furthermore, the LS method incorporates advanced mesh reinitialization capabilities, which can accurately describe the grain morphology of second-phase particle pinning and large deformation that most finite element models cannot handle [11,26,33,34].

Because the LS method can describe the local evolution of microstructure at a polycrystalline scale accurately, more and more scholars have studied mesoscale modeling under the LS framework. Bernacki et al. [35,36] accurately described the nucleation and growth processes of the polycrystalline materials GG and DRX based on the LS method and compared them with the JMAK theory. Hallberg [37] employed the LS method in conjunction with a finite element mesoscale model to investigate the recrystallization process in pure copper, providing an accurate description of dislocation density gradients within grains. It describes how the density of dislocations changes over a certain distance in the material’s microstructure. Scholtes et al. [38] introduced the modeling of microstructure evolution on the polycrystalline scale and accurately simulated the Zener pinning phenomenon without considering the shape of the second-phase particles. Maire et al. [26] applied the LS method to FF modeling of DRX and post-DRX (PDRX) in three-dimensional polycrystalline materials to simulate the grain topological evolution and recrystallization processes. Fausty et al. [34] proposed a new GG formula with heterogeneous grain boundary energy and found that the non-oriented distribution of the grain boundary was negatively correlated with the grain boundary function of energy. Murgas et al. [33] compared the performance of two finite element level set (FE-LS) formulas in 316 L stainless steel GG modeling, including the grain size, average value, and histogram.

Based on the above analysis, most of the current studies on DRX microstructure evolution models focus on describing dynamic recrystallization behavior through mathematical models. However, they are unable to capture the effects on the material’s microstructure or handle large deformation. Few researchers have conducted intuitive studies on mesoscale microstructure evolution and grain boundary motion during DRX. In this study, the Yoshie–Laasraoui–Jonas model based on the LS method was used to investigate the DRX microstructure evolution of GCr15 bearing steel. This approach enables a more accurate description of the effects of DRX behavior on microstructure evolution and grain boundary motion. Hot compression experiments were conducted using a Gleeble-1500D thermal simulator. A dislocation dynamics model for GCr15 was established based on the experimental flow stress data, and its model parameters were accurately identified using an inverse optimization method. The LS method was employed to predict microstructural evolution during the thermomechanical forming process under different hot processing parameters for GCr15 steel. The aim was to achieve prediction and control of material microstructural evolution through finite element simulations, which is crucial for improving GCr15 steel bearing components’ performance and optimizing their forging process designs.

## 2. Materials and Experiment

The material investigated in this study is GCr15 bearing steel, whose chemical composition is detailed in Table 1. Cylindrical specimens measuring ø8 × 12 mm were subjected to uniaxial hot compression using a Gleeble-1500D thermal simulator (Dynamic Systems Inc. (DSI), Poestenkill, NY, USA). Real-time measurement of sample temperature was conducted using a thermocouple sensor, and closed-loop temperature control was enabled for hot compression.

**Figure 1:**
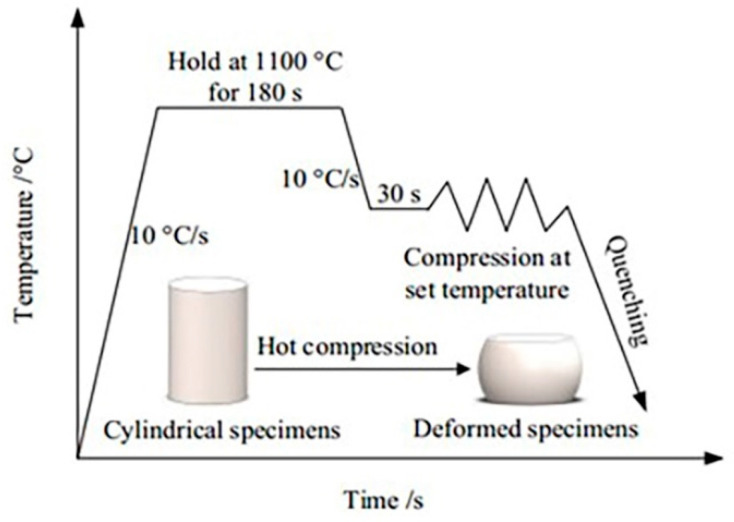
Hot compression test process diagram.

The experimental procedure is illustrated in Figure 1. The deformation temperature for GCr15 steel ranges from 850 to 1050 °C, with strain rates of 0.01 to 5 s^−1^. The cylindrical samples were heated to 1100 °C at a rate of 10 °C/s and held at that temperature for 180 s to ensure complete austenitization. The samples were then cooled to the target deformation temperature at a rate of 10 °C/s and held for 30 s to achieve uniform temperature distribution. Subsequently, single-pass compression was performed at the corresponding strain rate, with a maximum compression of 50%. After compression, the cylindrical samples were immediately water-cooled to preserve the microstructure state at the end of DRX.

## 3. Modeling of Dynamic Recrystallization for GCr15 Steel

### 3.1. LS Modeling of DRX

**Figure 2:**
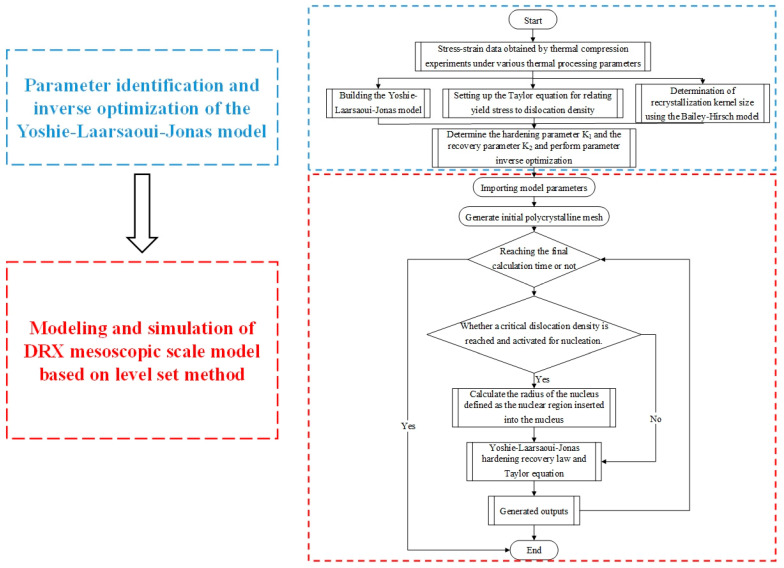
The whole process of DRX microstructure evolution modeling of GCr15 steel.

In the proposed DRX model based on the LS method, three reasonable assumptions were made, referencing previous studies, to ensure a simulation closer to the actual deformation process while reducing computational cost [26]: (1) The initial dislocation density within each grain is uniformly distributed, with no dislocation density gradient. (2) The average dislocation density of grains in the RVE is assumed to evolve according to the Yoshie–Laasraoui–Jonas law. During grain boundary migration, the scanned region is assumed to be nearly defect-free, i.e., dislocation-free. (3) The effects of second-phase particles and other internal defects within the grains are ignored, and dynamic recrystallization nucleation is assumed to occur only at the grain boundaries, consistent with the grain boundary bulging nucleation mechanism. Figure 2 illustrates the entire process of establishing the microstructure evolution model for the DRX process of GCr15 steel, combining the LS method and the Yoshie–Laasraoui–Jonas dislocation dynamics model.

#### 3.1.1. Level Set Method Formulation

**Figure 3:**
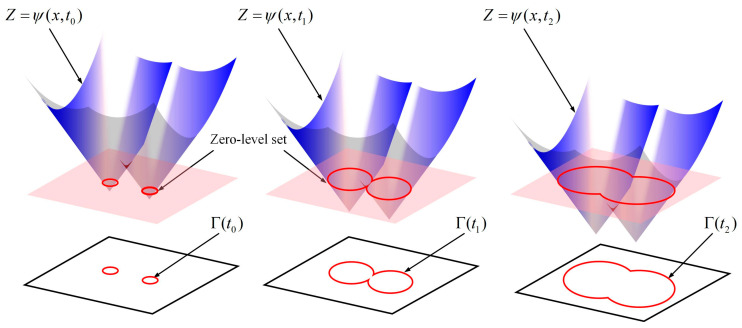
LS function and zero-level outline.

In 1988, Osher and Sethian proposed the LS method [39] to describe interface curvature tracking and evolution; this method has been widely used in DRX research. As shown in Figure 3, the LS method defines the closed curve formed by a two-dimensional plane on a three-dimensional surface as *Γ* and identifies it as the zero-level contour of the LS function *ψ*. By tracking numerous physical and chemical processes of the interface evolution of the LS function *ψ*, the evolution and tracking of the space curve *Γ* are realized.

The LS function *ψ* is defined as the symbolic distance function from the subdomain in the domain *Ω* to the closed curve *Γ* with the following implicit expression [40]:

(1) $$\left\{\begin{array}{l} \psi (x,t)=\pm d(x,\Gamma ),x\in \Omega \\ \Gamma (t)=x\in \Omega ,\psi (x,t)=0 \end{array}\right.$$

where *ψ* is the node coordinates in the domain *Ω* and *d* is the Euclidean distance.

The node state can be determined by the value of *ψ* at different nodes as shown in Equation (2):

(2) $$\left\{\begin{array}{ll} \psi (x)=-d(x,\Gamma ) & x\in \Omega_{-}\\ \psi (x)=+d(x,\Gamma ) & x\in \Omega_{+}\\ \psi (x)=0 & x\in \Gamma \end{array}\right.$$

**Figure 4:**
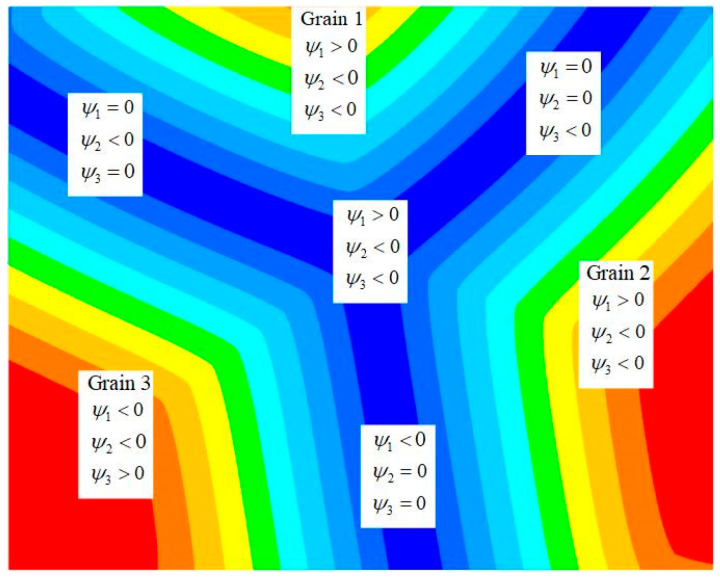
Interface description of the LS function.

The sign convention is used to define ψ>0 inside the grain and ψ<0 outside the grain. As shown in Figure 4, the LS method is used to describe the grain boundaries in an unstructured finite element mesh. Theoretically, each grain has its own LS function, but this is too computationally demanding for a computer. To minimize the number of LS functions and reduce the computational cost, a coloring/re-coloring technique was developed so that two adjacent grains always have different colors, with each color representing an LS function.

On the mesoscopic scale, for each signed distance function, the kinematic laws of grain boundaries can be expressed as:

(3) $$\vec{\nu }=M_{b}(e-\gamma k)\vec{n}$$

where $\vec{\nu }$ represents the rate of grain boundary migration y, *M*_b_ represents the grain boundary mobility, *γ* represents the grain boundary energy, *k* represents the grain boundary curvature, *e* represents the stored energy, and $\vec{n}$ represents the outward normal unit vector. Boundary curvature describes the geometric curvature of grain boundaries or interfaces between crystals in a material. It plays a significant role in driving processes such as grain growth and recrystallization, as it is directly linked to the reduction in interfacial energy.

#### 3.1.2. Dynamic Recrystallization Grain Growth Mechanism

In the case of DRX, due to the influence of capillary effects and the gradient of grain boundary stored energy, grain boundaries undergo migration. The convection equation, described by solving Equation (4), is used to describe the evolution of the LS function for the grain boundary.

(4) $$\left\{\begin{array}{l} \frac{\partial \psi (x,t)}{\partial t}+\vec{v}\cdot \nabla \psi (x,t)\\ \psi (x,t=0)=\psi_{0}(x) \end{array}\right.$$

The grain boundary velocity field incorporates capillary effects, which reduce the total boundary surface area to minimize grain boundary energy, and stored energy gradient effects, where grains with higher internal energy are consumed by others, thereby lowering the system’s overall energy. This is defined as follows:

(5) $$\vec{\nu }={\vec{\nu }}_{e}+{\vec{\nu }}_{c}$$

(6) $${\vec{\nu }}_{c}=-M_{b}\gamma_{b}\Delta \psi \nabla \psi$$

(7) $${\vec{\nu }}_{e}=M_{b}[|E_{0}|]\nabla \psi$$

(8) $$M_{b}=M_{0}\exp(\frac{-Q_{m}}{R_{g}T})$$

where ${\vec{\nu }}_{c}$ is the grain boundary velocity field considering capillary effects, ${\vec{\nu }}_{e}$ is the grain boundary velocity field considering the gradient of grain boundary stored energy, $\gamma_{b}$ is the grain boundary energy, $\left[|E_{0}|\right]$ is the stored energy due to the accumulation of dislocations at the grain boundary, *M*_0_ is the pre-factor, *Q*_m_ is the activation energy for grain boundary migration, *R*_g_ is the universal gas constant, and *T* is the absolute temperature.

The Read–Shockley-type grain boundary energy function is currently the most commonly used model for low-angle grain boundaries [41], as shown in Equation (9).

(9) $$\gamma (\theta )=\left\{\begin{array}{l} \gamma_{max}\left(\frac{\theta }{\theta_{max}}\right)\left(1-\ln\frac{\theta }{\theta_{max}}\right),\theta <\theta_{max}\\ \gamma_{max},\theta \geq \theta_{max} \end{array}\right.$$

where *θ* represents the misorientation angle of the grain boundary and *θ_max_* is the limit between small-angle and large-angle grain boundaries, typically set at 15°.

#### 3.1.3. Nucleation Mechanism

The DRX nucleation is due to the accumulation of dislocation density within the material reaching a critical level, whereas universal dislocation density accumulation occurs at grain boundaries [9]. The critical dislocation density model used in this paper is deduced by the critical dislocation density equation of Roberts and Ahlblom [42], combining phenomenological laws and the LS method to define the nucleation phenomenon, as shown in Equation (10).

(10) $$\rho_{\mathrm{cr}}={\left[\frac{-2\gamma_{b}\dot{\varepsilon }\frac{k_{2}}{M_{b}\delta \tau^{2}}}{\ln(1-\frac{k_{2}}{k_{1}}\rho_{\mathrm{cr}})}\right]}^{1/2}$$

where *ρ*_cr_ represents the critical dislocation density, τ is the line energy of dislocations, and δ is a material parameter.

The critical nucleation radius for DRX is defined by the Bailey–Hirsch criterion [27]. It is defined to avoid the disappearance of DRX nuclei due to capillary effects. It is assumed that the nucleation occurs in the form of circular particles. Equation (11) represents this criterion.

(11) $$r^{*}=\omega \frac{2\gamma_{b}}{\rho_{\mathrm{cr}}\tau }$$

The nucleation rate $\dot{V}$ represents the probability of DRX nucleation occurring per unit time. It is described using the modified Peczak and Luton proportional nucleation model, as shown in Equation (12).

(12) $$\dot{V}=K_{g}\Phi \Delta t.$$

where *K*_g_ is the nucleation probability coefficient and Φ is the grain boundary area or total volume of the grain at $\rho_{i}>\rho_{\mathrm{cr}}$.

### 3.2. Dislocation Dynamics Model and Parameters Identification

During the hot deformation of metallic materials, dislocation density accumulates due to work hardening, while dynamic recovery leads to the partial annihilation of some dislocations. In this study, the Yoshie–Laasraoui–Jonas dislocation dynamics model [43] is adopted to define the evolution of the average dislocation density field during the hot deformation process, considering the evolution of *k*_1_ and *k*_2_. This is represented by Equation (13).

(13) $$\frac{\partial \rho }{\partial \varepsilon }=k_{1}-k_{2}\rho$$

where *ε* represents the strain, *k*_1_ is the work-hardening coefficient, and *k*_2_ is the dynamic recovery coefficient.

During the plastic deformation stage, the Taylor equation can express the relationship between the flow stress and the average dislocation density, as shown in Equation (14).

(14) $$\sigma =\sigma_{0}+M_{T}\alpha_{T}\mu b\sqrt{\rho }$$

where *σ* represents the flow stress, *σ*_0_ is the yield stress, *M*_T_ is a material constant typically taken as 3 for face-centered cubic (FCC) and body-centered cubic (BCC) metals, $\alpha_{T}$ is a constant (0.1–0.4) that accounts for the microstructural induced mode changes [44], *b* is the Burgers vector length [45], *ρ* represents the average dislocation density, and *μ* is the shear modulus.

When ε=0 and $\rho =\rho_{0}$, integrating Equation (13) and substituting it into Equation (14), the Taylor equation for the flow stress during the early stage of the hot deformation process can be obtained as shown in Equation (15).

(15) $$\sigma =\sigma_{0}+M_{T}\alpha_{T}\mu b\sqrt{(\rho_{0}-\frac{k_{1}}{k_{2}})\exp(-k_{2}\varepsilon )+\frac{k_{1}}{k_{2}}}$$

When ε tends to infinity, the flow stress can be approximated as the dynamic recovery saturation stress $\sigma_{\mathrm{sat}}$. In this case, the relationship between *k*_1_ and *k*_2_ can be expressed as follows:

(16) $$k_{1}={\left(\frac{\sigma_{\mathrm{sat}}-\sigma_{0}}{M_{T}\alpha_{T}\mu b}\right)}^{2}k_{2}.$$

Defining $\theta =\sigma_{0}-\sigma$ as the stress hardening value, Equation (17) can be derived.

(17) $$\theta \frac{\partial \theta }{\partial \varepsilon }=\frac{\theta_{\mathrm{sat}}^{2}}{2}k_{2}-\frac{k_{2}}{2}\theta^{2}$$

where the steady-state stress hardening value is specified as $\theta_{\mathrm{sat}}=\sigma_{\mathrm{sat}}-\sigma$.

**Figure 5:**
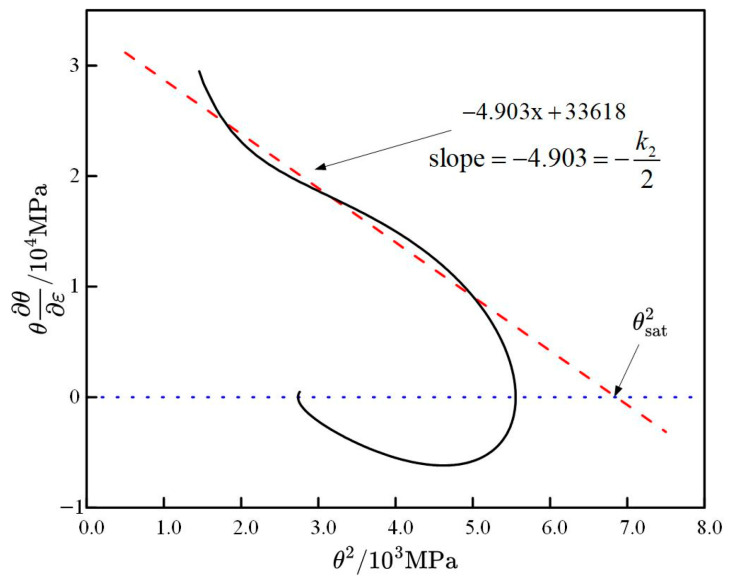
Fitted curve trend of $\theta \frac{\partial \theta }{\partial \varepsilon }-\theta^{2}$.

By substituting hot compression test data into Equation (17), the $\theta \frac{\partial \theta }{\partial \varepsilon }-\theta^{2}$ curve trend can be plotted. Taking the deformation rate of 0.1 s^−1^ at the deformation temperature of 1000 °C as an example, as shown in Figure 5, the deformation curve’s first 20% strain is too small, and sometimes the fitted slope may be positive, making the trend unsuitable for linear fitting. Furthermore, to reduce the influence of DRX stress softening effects, the deformation curve should be truncated at 80% of the peak strain. The dynamic softening coefficient *k*_2_ is twice the curve slope, and the intersection of the slope extension line with the x-axis is $\theta_{\mathrm{sat}}^{2}$.

**Figure 6:**
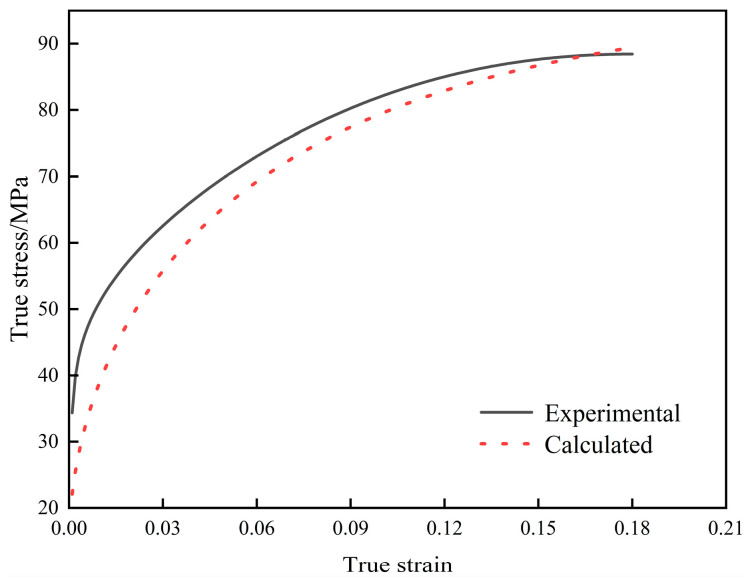
Comparison between the flow stress curve obtained by the test and the curve given by the model.

The work-hardening coefficient *k*_1_ can be determined using the *k*_2_ and $\theta_{\mathrm{sat}}^{2}$ obtained from Equation (16). By substituting *k*_1_, *k*_2_, *ρ*_0_, and *σ*_0_ into Equation (15), the calculated values of flow stress can be obtained. By comparing the calculated values from the dislocation dynamics model with the experimental data, as shown in Figure 6, it is evident that the predicted flow stress curve aligns relatively well with the experimental results, although some discrepancies still exist.

**Figure 7:**
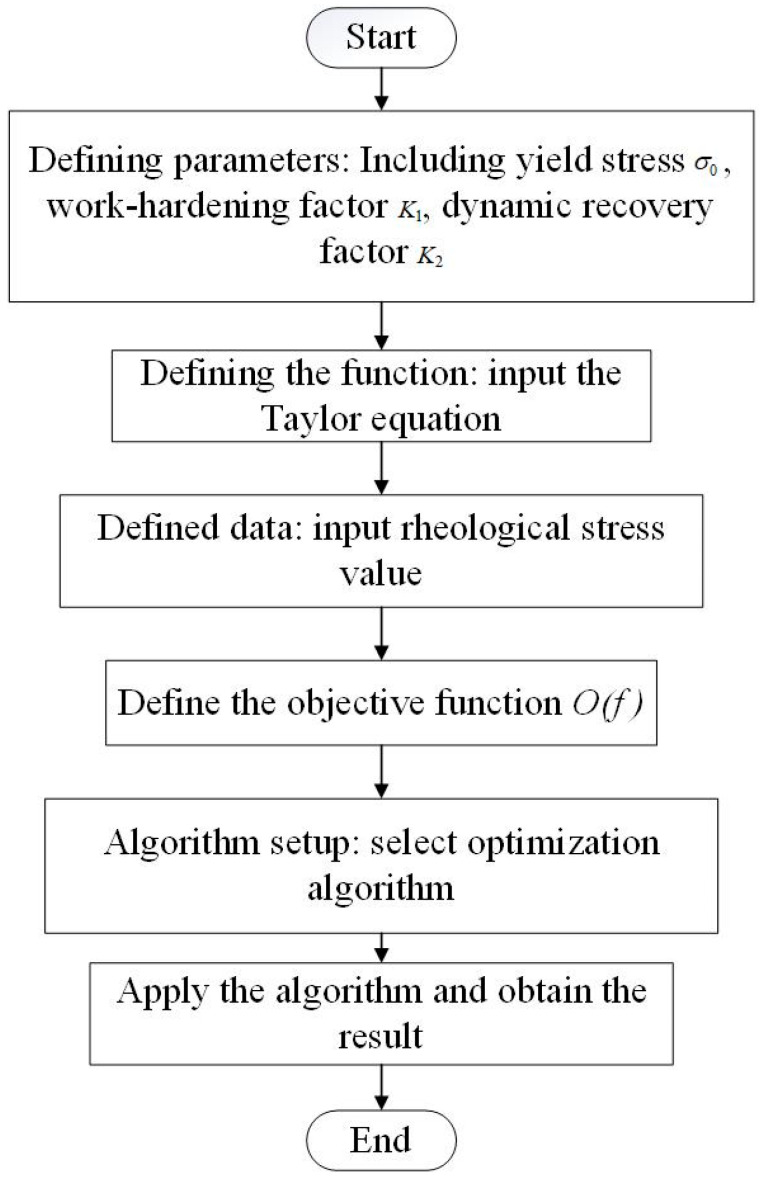
Inverse optimization process flowchart for model parameters.

To reduce the accumulation of errors, the three material parameters *k*_1_, *k*_2_, and *σ*_0_ are further optimized using the particle swarm optimization (PSO) algorithm to improve the model’s accuracy, thereby enhancing the prediction accuracy [46]. PSO was selected for this study due to its simplicity, efficiency, and effectiveness in handling complex, non-linear optimization problems. PSO has demonstrated strong global search capabilities, and it converges efficiently for the type of multi-dimensional parameter optimization required in this study. The optimization process is shown in Figure 7. The mean squared error between the calculated and tested flow stress values is used as the objective function, as shown in Equation (18).

(18) $$O(f)=\frac{\sum_{i}^{n}{\left(\sigma_{i}^{\exp}-\sigma_{i}^{\mathrm{cal}}\right)}^{2}}{\sum_{i}^{n}{\left(\sigma_{i}^{\exp}\right)}^{2}}$$

where $\sigma_{i}^{\exp}$ is the *i*th tested stress value, and $\sigma_{i}^{\mathrm{cal}}$ is the *i*th calculated stress value.

In order to better analyze the accuracy of the stress–strain curves before and after optimization, the predicted values of the model parameters before and after optimization are listed in Table 2 for comparison.

**Figure 8:**
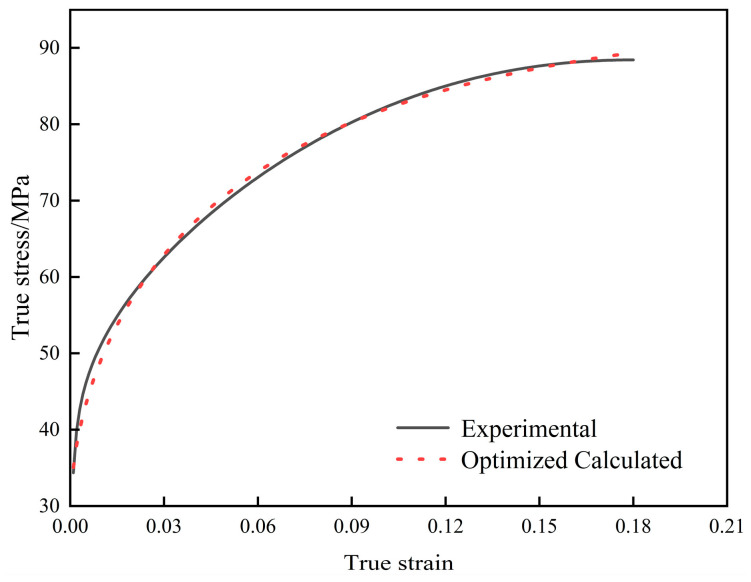
Comparison of flow stress curves after optimization and experimental curves.

Figure 8 shows a comparison of the flow stress curves during the work-hardening and recovery stages, calculated using the dislocation dynamics model after inverse optimization, with the experimental values. The results demonstrate that applying inverse optimization significantly improves the model’s accuracy, thereby enhancing its predictive capability.

**Figure 9:**
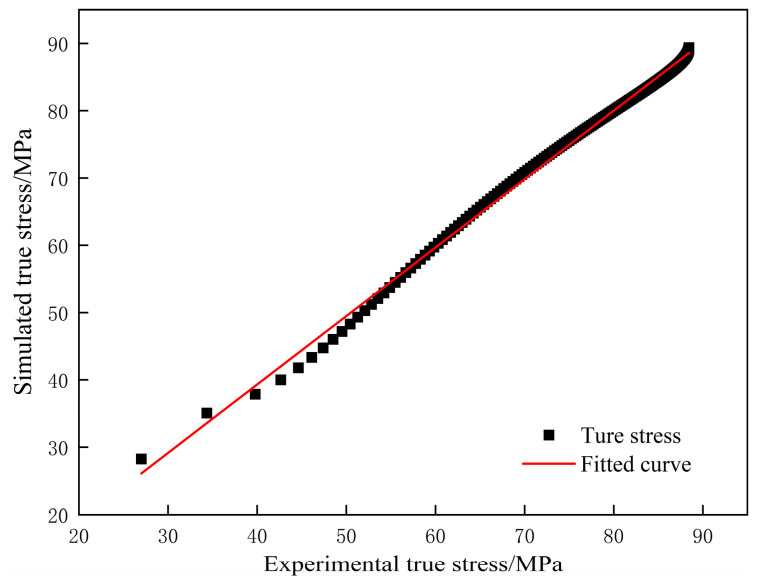
Comparison of experimental and simulated flow stress values.

To assess the accuracy of the optimized dislocation dynamics model predictions using the correlation coefficient (*R*), as shown in Figure 9, the computed value for *R* is 0.9969. This indicates that the constructed model effectively predicts the flow stress behavior of GCr15 steel.

### 3.3. Mesoscale Finite Element Modeling Based on Level Set Method

**Figure 10:**
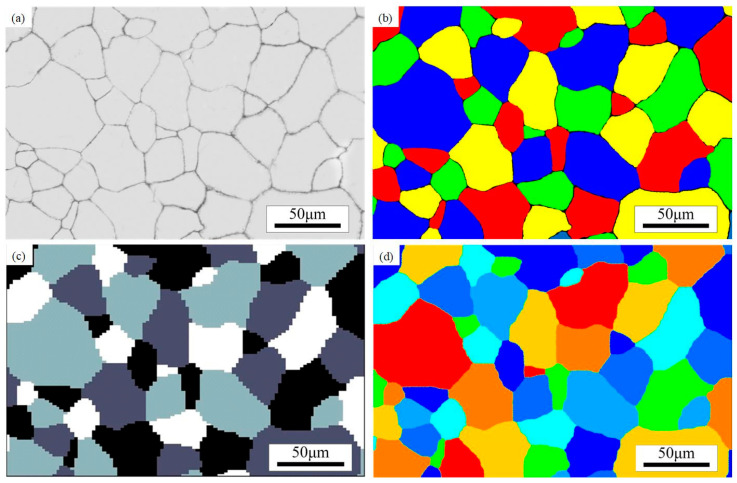
Initial grain in situ import diagram: (**a**) initial grain matrix, (**b**) initial grains after coloring, (**c**) initial grains after segmentation, and (**d**) initial grain model based on LS method.

This text uses the commercially available full-domain simulation software DIGIMU4.0^®^ for simulation. It is specifically designed for modeling GG and DRX. Figure 10 shows the initial grain finite element model.

The initial grain finite element model was imported in situ based on the original metallographic image. Figure 10a shows the matrix formed by the original metallographic image. Image processing techniques were used to color the original metallographic image (Figure 10b), with the coloring principle being to minimize the number of colors while ensuring that adjacent grains had different colors. The colored grains were divided into 100 × 100 slices, with the horizontal and vertical coordinates of each slice associated with the corresponding color, and written into the DIGIMU^®^ finite element simulation software for computation, as illustrated in Figure 10c. The resulting initial grain finite element model is shown in Figure 10d. Comparing Figure 10a and Figure 10d, the high similarity between them demonstrates that this method can effectively achieve in situ importation of the original metallographic image.

**Figure 11:**
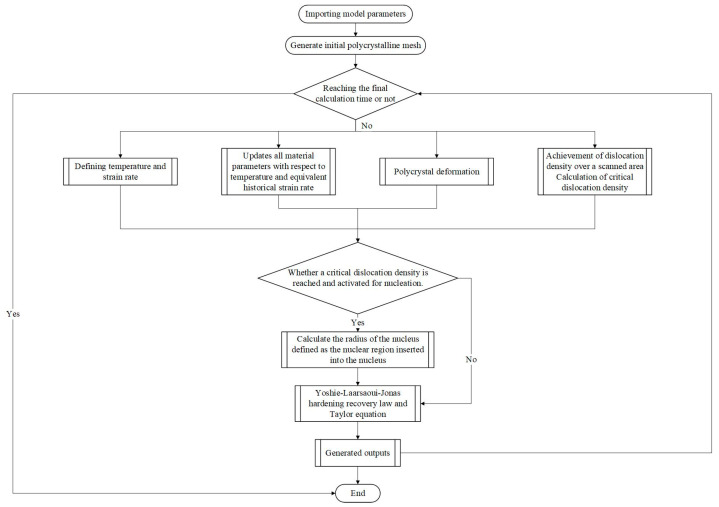
Flowchart of DRX simulation calculation using the LS method.

Figure 11 shows a flowchart of DRX simulation using the LS method. The initial grain finite element model and mesh structure are generated from the original metallographic image. We determined whether the final deformation time had been reached based on the defined thermoforming parameters, and we then proceeded with polycrystalline deformation. By scanning the dislocation density at each grain boundary, the critical dislocation density was calculated, and it was determined whether nucleation occurred. The LS method describes the occurrence of GG and DRX through the strong evolution of microstructural topology during thermomechanical deformation, coupling the complex competition among boundary migration, recovery, and nucleation.

## 4. Results and Discussion

### 4.1. Analysis of the Flow Stress Curves

**Figure 12:**
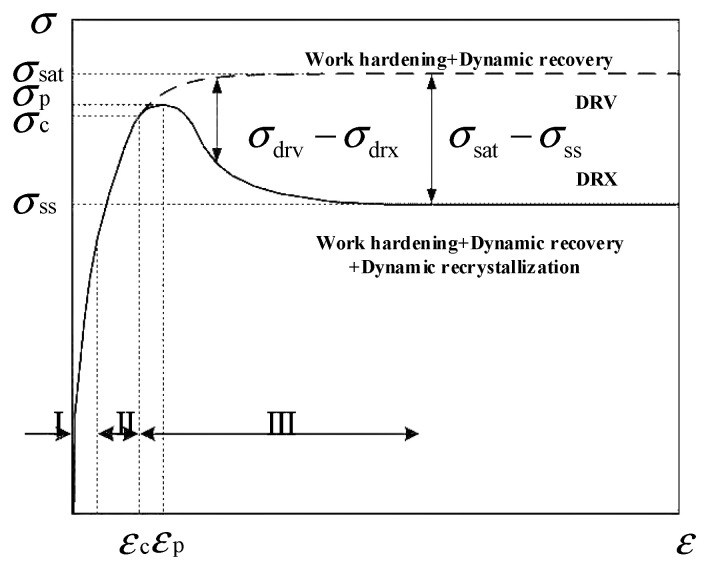
Typical flow stress curves during high-temperature deformation.

During the hot deformation process of metallic materials, the microstructural evolution mechanisms at different stages can significantly influence the macroscopic performance indicators, which are well reflected in the flow stress curves. As shown in Figure 12, two typical types of flow stress curves are observed during the isothermal compression deformation of metallic materials. In Figure 12, the solid line represents the curve with dynamic recrystallization characteristics, while the dashed line represents the typical dynamic recovery curve. Regions I, II, and III in Figure 12 correspond to the work hardening, dynamic recovery, and the stages of dynamic recovery combined with dynamic recrystallization during the hot deformation process, respectively. The dynamic recovery-type curve shows that as the strain increases, the flow of stress gradually rises and eventually stabilizes, reaching a saturation stress. In contrast, the dynamic recrystallization-type curve demonstrates that as the strain increases, the flow stress reaches a peak value and then gradually decreases, finally stabilizing at a steady-state stress.

The flow stress during hot processing is considered a crucial foundation for predicting the behavior of microstructural evolution and grain boundary migration, as well as for understanding the relationship between the mechanical properties of the material and its microstructure. Additionally, different hot processing parameters can be formulated based on the material’s flow stress curve to provide corresponding process optimization schemes. The material coefficients and physical constants used in this simulation are shown in Table 3.

**Figure 13:**
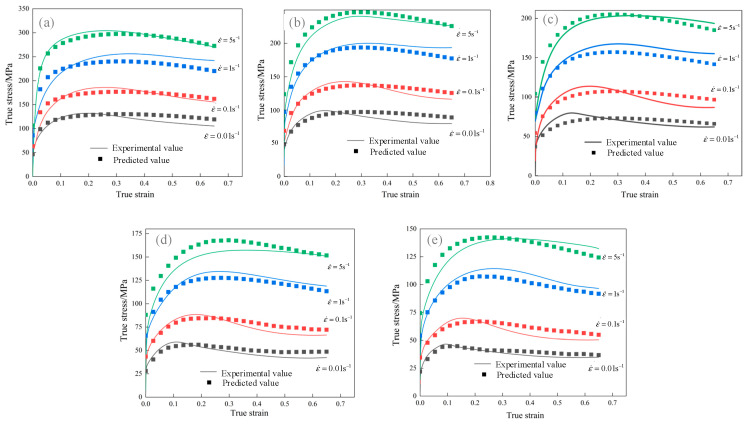
Comparison between the test value and the value predicted by the LS model at the following temperatures: (**a**) 850 °C, (**b**) 900 °C, (**c**) 950 °C, (**d**) 1000 °C, and (**e**) 1050 °C.

The typical features of the DRX rheological stress curve reflect the microstructural evolution and mechanical behavior of the material. The LS model was employed to predict the stress–strain curve across various deformation conditions, with a comparison to the experimental data presented in Figure 13. In summary, the predicted and experimental values show good agreement, both displaying distinct single-peak DRX curve features. This shows that the LS model can accurately predict the hot deformation process of GCr15 steel. However, there is still some discrepancy between the tested and predicted values during the work-hardening recovery stage. This discrepancy arises because different regions of the hot compression specimen experience varying degrees of DRX, a difference that is neglected in the LS model simulation.

### 4.2. Simulation and Verification of Microstructure Evolution for GCr15 Steel

#### 4.2.1. Effects of Strain on DRX Behavior

**Figure 14:**
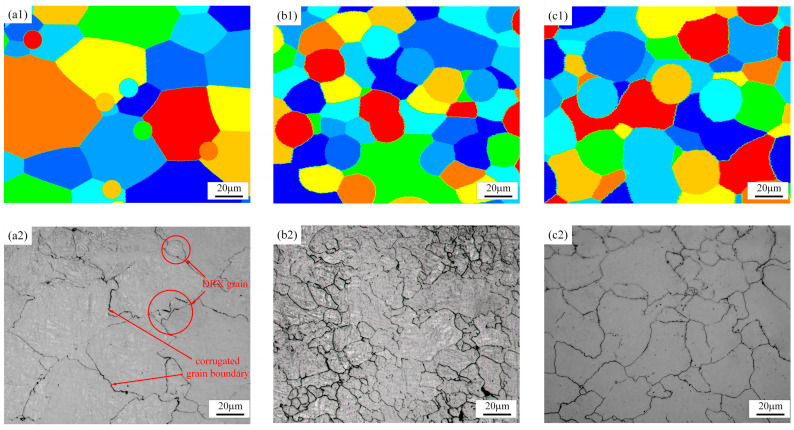
Comparison between LS model simulation results and experimental results at 1000 °C/0.1 s^−1^. (**a1**,**a2**) Strain of 0.14, (**b1**,**b2**) strain of 0.42, and (**c1**,**c2**) strain of 0.7.

The DRX microstructure evolution process of GCr15 steel under different amounts of strain during thermal compression deformation at a deformation temperature of 1000 °C and a strain rate of 0.1 s^−1^ is shown in Figure 14. Figure 14a1,a2 compares the microstructure at a strain of 0.14 between the LS method predictions and experimental values. It can be seen that at a strain of 0.14, the critical dislocation density has been reached, and DRX grains begin to appear, although the recrystallization rate is still low at this stage. The newly formed nuclei are small, dislocation-free crystals. The initiation of nucleation is usually related to the migration of grain boundaries, driven by the stored energy from dislocations and grain boundaries generated during previous deformation. When the deformed matrix reaches the critical dislocation density, nucleation or defects can occur at the matrix or grain boundaries. This is highlighted in Figure 14a2, where irregularly arranged recrystallized grains and wavy grain boundaries appear between grains. As the strain increases, the dislocation density and deformation energy of the material increase, resulting in a decrease in DRX grain size and an increase in the number of DRX grains. Secondary recrystallization occurs when the material once again reaches the critical dislocation density. As the deformation amount further increases, as shown in Figure 14b1,b2, the degree of recrystallization is significantly greater than at lower strains, with recrystallized grains occupying most of the area, although a few large, uncrystallized original grains can still be seen. As the strain continues to increase, as shown in Figure 14c1,c2, when the strain reaches 0.7, the degree of recrystallization further increases, and the material has essentially completed DRX. The results of simulation calculations using the LS method are in good agreement with the metallographic test results.

**Figure 15:**
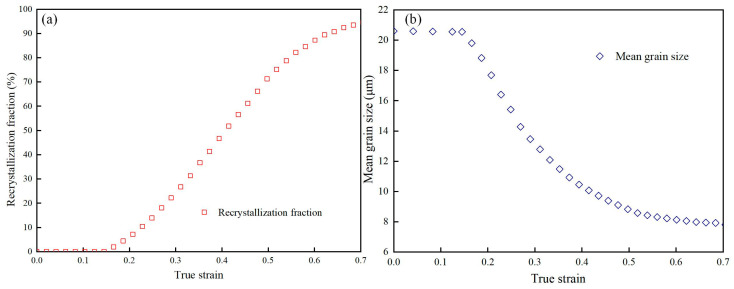
The simulated values of the DRX fraction (**a**) and the mean grain size (**b**) at 1000 °C/0.1 s^−1^.

It can be seen from Figure 15 that with the increase in the degree of deformation, the DRX volume fraction initially increases rapidly, then increases slowly, and finally gradually stabilizes. The mean grain size first decreases sharply, then decreases slowly, and finally stabilizes, with periodic nucleation occurring throughout this process. At a strain of 0.14, DRX has just started, and only partial recrystallization can be observed, with a DRX fraction of only 1.14% and a mean grain size of 20.55 µm. As the strain increases to 0.42, the DRX fraction increases to 53.13%, and the mean grain size is 9.21 µm. When the strain reaches 0.7, the DRX fraction is 98.09%, and the mean grain size is 7.37 µm. At this point, further increasing the strain has no significant effect on the DRX parameters. In summary, DRX can significantly reduce grain size, achieving the effect of grain refinement.

**Figure 16:**
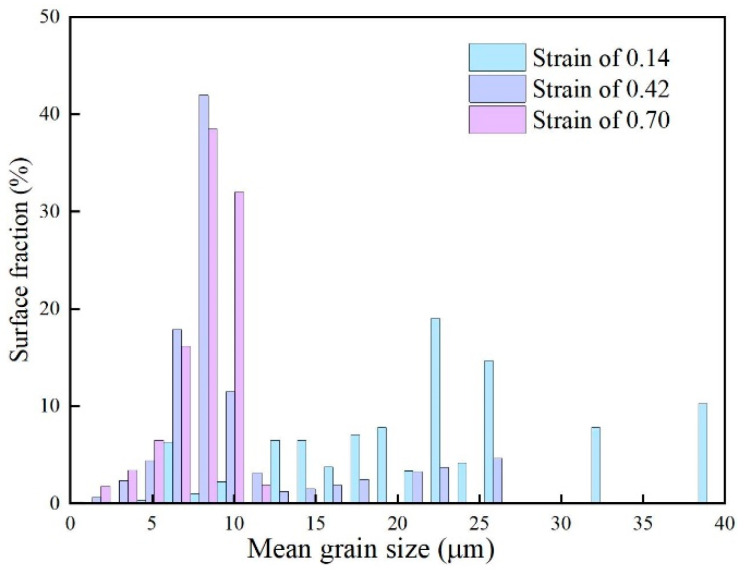
Surface-weighted grain size distribution at 1000 °C/0.1 s^−1^.

The effect of different strains on DRX can also be visually represented using distribution maps. Figure 16 compares the surface-weighted grain size distribution under different strain conditions. As shown in Figure 16, with increasing strain, the average grain size in the microstructure gradually decreases. This is in agreement with previous simulations and tested results.

#### 4.2.2. Effects of Temperature on DRX Behavior

**Figure 17:**
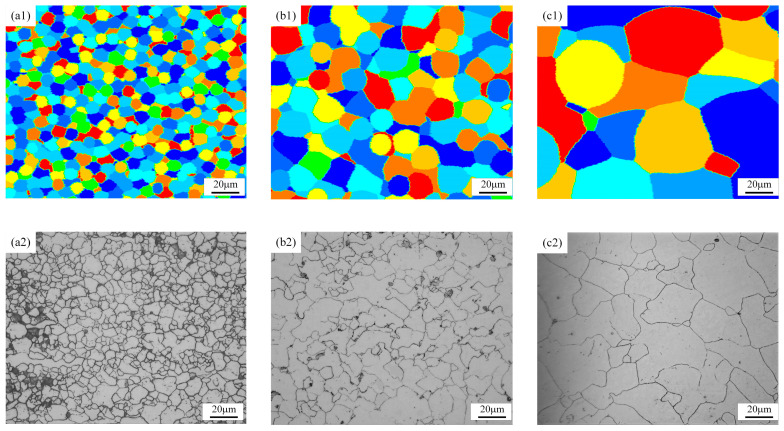
Comparison between LS model simulation results and experimental results at 0.1 s^−1^/0.7. (**a1**,**a2**) Temperature of 850 °C, (**b1**,**b2**) temperature of 950 °C, and (**c1**,**c2**) temperature of 1050 °C.

Figure 17 compares the grain morphology of GCr15 steel under different deformation temperatures (850, 950, and 1050 °C) at a strain rate of 0.1 s^−1^ and a compression ratio of 50%, using LS model simulation results and metallographic tests. As shown in Figure 17, under certain other hot deformation parameters, recrystallization at 850 °C is already very complete, with uniform grain size and equiaxed grains. The grains at 950 °C are larger than those at 850 °C, while the grains at 1050 °C are even coarser. The results show that under the same deformation conditions, as the temperature increases, the grain size also increases, leading to coarser grains. This phenomenon is partly due to the increased dislocation movement within the grains caused by higher deformation temperatures, which results in sufficient grain boundary migration. This intensifies dislocation slip and atomic diffusion, thereby increasing the recrystallization nucleation rate [47], resulting in small grains being swallowed by large grains before they grow larger, which causes the microstructure to coarsen.

**Figure 18:**
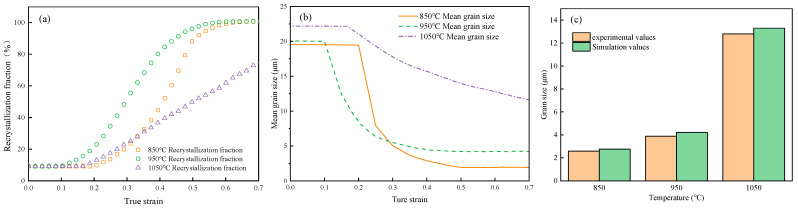
Under the condition of 0.1 s^−1^/0.7, (**a**) the DRX fraction, (**b**) the simulated average grain size, and (**c**) a comparison of grain size between the simulation and the experiment are shown.

From Figure 18a, the recrystallization fraction of GCr15 steel increases as the temperature decreases, with the recrystallization fractions at 850 °C and 950 °C reaching 100%, while the recrystallization fraction at 1050 °C is only 71.065%. Figure 18b shows that at 850 °C and 950 °C, due to complete recrystallization, the mean grain size decreases significantly. However, at 1050 °C, due to incomplete DRX, the mean grain size decreases only slightly, by just 9.1 µm. From the comparison of simulated and experimental mean grain sizes in Figure 18c, it can be observed that the calculated results are in good agreement with the experimental results.

#### 4.2.3. Effects of Strain Rate on DRX Behavior

**Figure 19:**
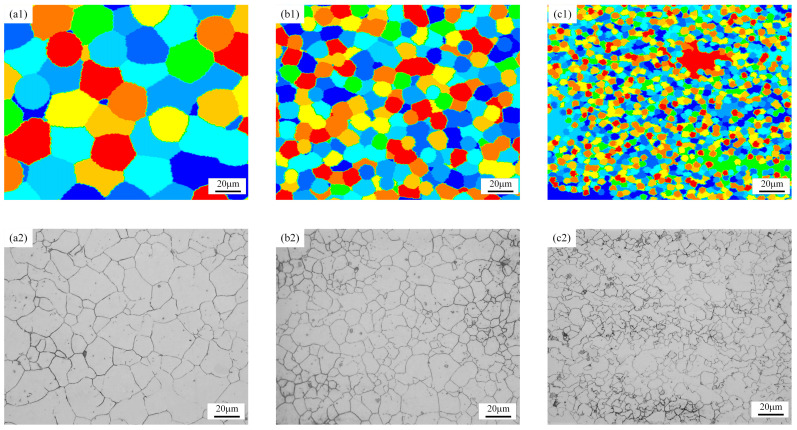
Comparison of LS model simulation results and test results at 900 °C/0.7 for strain rates of (**a1**,**a2**) 0.01 s^−1^, (**b1**,**b2**) 0.1 s^−1^, and (**c1**,**c2**) 1 s^−1^.

To study the effect of strain rate on DRX behavior, Figure 19 shows the evolution of DRX grain morphology of GCr15 steel at a deformation temperature of 900 °C and a strain of 0.7, with strain rates of 0.01, 0.1, and 1 s^−1^, obtained through simulation and how they compare with experimental results. As the strain rate increases, the higher strain rate leads to insufficient growth time for recrystallization nuclei, resulting in a finer grain structure. The results indicate that the simulated microstructure morphology aligns well with the metallographic test results overall. However, certain discrepancies between experimental and simulated values remain. The reason for these differences is that the current model does not account for the influence of second-phase particles on dynamic recrystallization (DRX). These particles can significantly impact the nucleation and grain growth processes through mechanisms such as Zener pinning, potentially altering the microstructural evolution.

**Figure 20:**
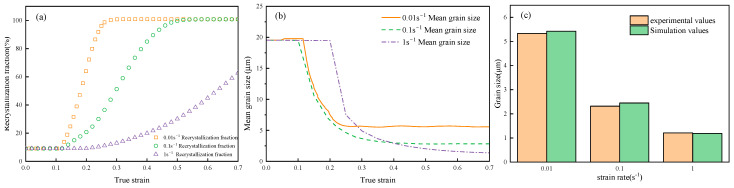
Under the condition of 900 °C/0.7, the following are illustrated: (**a**) DRX fraction, (**b**) simulated average grain size, and (**c**) a comparison of grain size between simulation and experiment.

Figure 20 presents the curves for the recrystallization fraction and mean grain size under different strain rates, with other hot deformation parameters held constant. From Figure 20a, it is evident that a lower strain rate leads to a smaller critical strain for DRX and a smaller strain required for complete DRX. At a strain rate of 1 s^−1^, due to the significant reduction in thermal forming time under high strain rates, the phenomenon of suppressed DRX fraction occurs, with a recrystallization fraction of 58.4%. Figure 20b shows that, under the same deformation conditions, as the strain rate increases from 0.01 s^−1^ to 1 s^−1^, the grain size gradually decreases from 5.42 µm to 1.19 µm, indicating that higher strain rates are conducive to obtaining fine-grained structures. This is because high strain rates reduce the deformation time of the specimen, decreasing the time for dislocation movement and grain boundary migration, which results in a higher amount of stored strain energy within the grains. Additionally, the dislocation density and accumulated strain energy within the grains increase with the strain rate, making nucleation at grain boundaries easier.

The DRX simulation results help identify the optimal combination of strain, strain rate, and temperature to promote desired microstructural characteristics, such as refined grains, which can improve mechanical properties such as hardness and wear resistance. By understanding the relationship between DRX behavior and microstructure evolution, manufacturers can better control grain size and dislocation density, directly influencing the fatigue life and reliability of GCr15 steel components in applications such as bearings.

## 5. Conclusions

The aim of this study is to effectively control and predict the microstructure of GCr15 steel. By combining hot compression tests with numerical modeling, a DRX microstructure evolution model for GCr15 steel was established based on the LS method within an FE framework. The microstructure evolution model was validated by comparing metallographic experiments with the simulation results of the microstructure evolution of GCr15 steel. The conclusions are as follows:

1. A Yoshie–Laasraoui–Jonas dislocation dynamics model was established using dislocation density as an internal variable, considering uniform grain boundary energy and mobility, and parameter identification was performed. To reduce cumulative errors, a particle swarm optimization algorithm was proposed for parameters’ inverse optimization. The optimized model’s correlation coefficient was 0.9969. This shows that the inverse optimization method for microstructure model parameters proposed in this paper is accurate and effective.
2. A DRX microstructure evolution model based on the LS method was established, and the Yoshie–Laasraoui–Jonas model parameters were embedded in DIGIMU^®^ software through secondary programming for simulation calculations. The initial grain morphology was reconstructed in situ to study its influence on microstructural topology. The established model was validated through metallographic experiments by comparing actual metallographic images with simulation results. It was demonstrated that the finite element microstructure evolution model can predict complex grain topology evolution after large and non-uniform deformations.
3. The impacts of different hot processing parameters on the DRX microstructure evolution of GCr15 steel were studied and analyzed using curve charts and distribution maps. The results were validated by comparing the hot compression experiment results with the simulation results. The findings indicate that hot deformation significantly affects grain size, with lower strain rates and higher temperatures both leading to a reduction in the critical strain for DRX, resulting in larger grain sizes.

Future research could incorporate the effects of second-phase particles and anisotropic grain boundary energies to further enhance the model’s predictive accuracy. The validated model could be extended to simulate industrial-scale hot deformation processes, such as forging and rolling, providing practical guidelines for optimizing processing parameters to achieve desired microstructures.

## Figures and Tables

**Table 1:** Chemical composition of GCr15 steel (wt.%).

C	S	P	Mn	Si	Cr	Mo	Cu	Ni	Fe
0.96	0.006	0.013	0.36	0.19	1.46	0.02	0.06	0.08	Balance

**Table 2:** Model parameters of GCr15 steel before and after optimization.

	*σ*_0_ (MPa)	*k*_1_	*k*_2_
Before optimization	34.352	1.195 × 10^9^	9.806
After optimization	35.063	8.334 × 10^8^	10.719

**Table 3:** Initial material parameters of GCr15 steel DRX simulation.

Symbol	Physical Meaning	Value
*M*_0_	Grain boundary mobility pre-factor/(mm^4^·J^−1^·s^−1^)	2.93 × 10^10^
*Q*_m_	Crystal boundary migration activation energy/(J·mol^−1^)	2.18 × 10^5^
μ	Shear modulus/(GPa)	80
*α*_T_	Constants for microstructure-induced pattern changes	0.2
*T*_m_	Melting temperature/(°C)	1400
b	Burr vector (geometry)/(mm)	2.86 × 10^−7^
*K*_g_	Nucleation probability coefficient	3
*γ*_b_	Grain boundary energy/(J·mm^−2^)	5.6 × 10^−7^
ω	Safety factor	1.5
*R*_g_	Gas constant/(J·mol^−1^·K^−1^)	8.314

## Data Availability

The data presented in this study are available on request from the corresponding author. The data are not publicly available due to these data being part of ongoing research.
